# Immune checkpoint inhibitors-associated cranial nerves involvement: a systematic literature review on 136 patients

**DOI:** 10.1007/s00415-024-12660-2

**Published:** 2024-09-03

**Authors:** Samuel Pichon, Pauline Aigrain, Charlotte Lacombe, Bruno Lemarchant, Emmanuel Ledoult, Vincent Koether, Amélie Leurs, Ghadi Zebian, David Launay, Benoit Gachet, Clémentine Levy

**Affiliations:** 1 Service de Médecine Interne, Centre de Référence des Maladies Auto-Immunes et Auto-Inflammatoires Systémiques Rares de L’Adulte du Nord, Nord-Ouest, Université de Lille, CHU Lille, Méditerranée et Guadeloupe (CeRAINOM), Pointe-à-Pitre, France; 2 Pôle de Réanimation, Université de Lille, CHU Lille, 59000 Lille, France; 3 Département de Neurologie, CRC-SEP, Université de Lille, CHU Lille, 59000 Lille, France; 4 U1286-INFINITE-Institute for Translational Research in Inflammation, INSERM, Université de Lille, CHU Lille, Lille, France; 5 Département de Médecine Interne et Maladies Infectieuses, CH Dunkerque, 59240 Dunkerqu, France; 6 Service de Maladies Infectieuses, CH Gustave Dron, Université de Lille, 59200 Tourcoing, France; 7 ULR 2694 METRICS Evaluation des Technologies de Santé et des Pratiques Médicales, CH de Tourcoing, Université de Lille, 59000 Lille, France

**Keywords:** Immune checkpoint inhibitors, Nivolumab, Pembrolizumab, Ipilimumab, Immune-related adverse events, Cranial palsy

## Abstract

**Objective:**

Describe the demographic data and clinical phenotype of cranial palsy induced by immune checkpoint inhibitors (CNP-ICI).

**Methods:**

A systematic literature review of the literature was performed in Pubmed, Web of Science, and Embase, including 68 articles and 136 patients (PROSPERO no. CRD42024517262).

**Results:**

Out of the 1205 articles screened, 68 articles were included after fulfilling the inclusion criteria, for a total of 136 patients. All articles were case reports and case series. In the cohort studied, 52% of patients were treated with anti PD-1/PDL-1 therapies, 14% with anti CTLA-4 therapies, and 34% with a combination of anti CTLA-4 and anti PD-1/PDL-1 therapies. The facial nerve was the most affected cranial nerve, involved in 38% of cases, followed by the optic nerve (35%), the cochleovestibular nerve (12%), and the abducens nerve (10%). The median time from the initial immune checkpoint inhibitor (ICI) injection to the onset CNP-ICI was 10 weeks (IQR 4–20). Magnetic resonance imaging demonstrated contrast enhancement or abnormal signal of the affected nerve in 43% of cases. Cerebrospinal fluid analysis indicated lymphocytic pleocytosis in 59% of cases. At the onset of immune-related adverse events, 89% of patients discontinued immunotherapy, and 92% received treatment for CNP-ICI. Treatment regimens included corticosteroids in 86% of cases, intravenous immunoglobulin in 21%, and plasma exchange in 5.1%. Among the whole population, 33% achieved recovery, 52% showed clinical improvement, 16% remained stable, and 3% experienced worsening of their condition. Rechallenge with immunotherapy was significantly associated with the emergence of new immune-related Adverse Events (irAEs).

**Conclusion:**

ICI therapy may lead to cranial nerve involvement, particularly affecting the facial nerve, typically presenting around 10 weeks after treatment initiation. While corticosteroid therapy often resulted in patient improvement, rechallenging with ICIs were associated with new irAEs.

**Supplementary Information:**

The online version contains supplementary material available at 10.1007/s00415-024-12660-2.

## Introduction

Immune checkpoint inhibitors (ICI) are increasingly used in oncology and have marked a therapeutic and prognostic revolution for patients suffering from cancers such as melanoma, and lung cancers [1–3]. ICI restores anti-tumor immunity by inhibiting the interaction between immune checkpoints such as programmed cell death protein-1 (PD-1) or cytotoxic T lymphocyte antigen-4 (CTLA-4) and their ligands. Because immunotherapies effects can be compromised by immune escape mechanisms, dual immunotherapy regimens have emerged to overcome this challenge by inhibiting both target [4].

While such treatments provide an overall tumor response, they are not without risk. Up to 70% of patients on anti-PD1/PDL-1 and 90% of patients treated with anti-CTLA-4 will experience immune-related adverse events (irAEs) [5]. These irAEs can potentially involve every organ system with a predominance of the skin, lung, endocrine and gastrointestinal systems [6]. Among the irAEs, neurologic immune-related adverse events are rare, they occur between 1 and 12% of the cases [5, 7, 8].

Among n-irAEs, cranial nerve palsy induced by ICI (CNP-ICI) are poorly described entity. A French multicenter retrospective study in 2020, with a systematic review of the literature, described the clinical characteristics of 39 CNP-ICI [9]. Numerous case reports and case series have since been published, possibly refining the spectrum of these toxicities.

The present study aims to describe the demographic data, clinical characteristics, management, and outcome of CNP-ICI through with a systematic literature review.

## Patients and methods

We performed a meta-analysis according to the Preferred Reporting Items for Systematic Reviews and MetaAnalyses (PRISMA) Statement protocol [10]. This study was declared on PROSPERO (CRD42024517262).

We included patients who presented with cranial neuropathy attributed to ICI. Neurological conditions such as myasthenia gravis or Guillain-Barré syndrome were included only if cranial nerve symptoms were described. We aimed to describe demographic data, clinical characteristics, and to describe and analyze the management of CNP-ICI. Outcome was defined by 4 categories: complete resolution, clinical improvement, stability of damage and experienced worsening. We included all experimental trials (randomized controlled), and observational studies (cohort, case–control, case series, case report) focusing on CNP-ICI. We excluded expert opinion, reviews, and pooled analyses.

We searched through published studies on PubMed, Web of Science and Embase databases until 31 December 31st 2023. We used combinations of the terms “palsy immune checkpoint inhibitors”, “neuritis immune checkpoint inhibitors”, “cranial nerve immune checkpoint inhibitors”, “neuropathy immune checkpoint inhibitors”. The search strategy was adapted to meet the specificities of each database. References of the included studies were systematically searched for any additional relevant articles and reviews were used to identify potential eligible articles. No language restriction was applied.

Then, two authors (S.P and P.A) independently reviewed and assessed the eligibility of titles and abstracts. The full text of the remaining articles, including the references, was examined to determine whether the articles contained relevant information. Any discrepancies were resolved through discussion with a third member of the review (C.L.). The process of study selection was documented in a PRISMA flow diagram.

For each paper, we extracted the following data: sex, age, tumor type, cranial nerve involved, others irAEs manifestations, ICI used, delay since first ICI to clinical onset, findings of cerebrospinal fluid and Magnetic Resonance Imaging (MRI) studies, onconeural antibody, treatment, outcome of IrAE and tumor, rechallenge and toxicity. When the data could not be individualized or some information was missing (e.g., the type of IC or the cranial nerve involved), we contacted the authors of the original papers.

Risk of bias was not assessed due to the higher number of case reports and series included.

### Quality assessment

Study quality was assessed using a tool for evaluating the methodologic quality of case reports and case series proposed by Murad et al. [11].

## Results

**Fig. 1:**
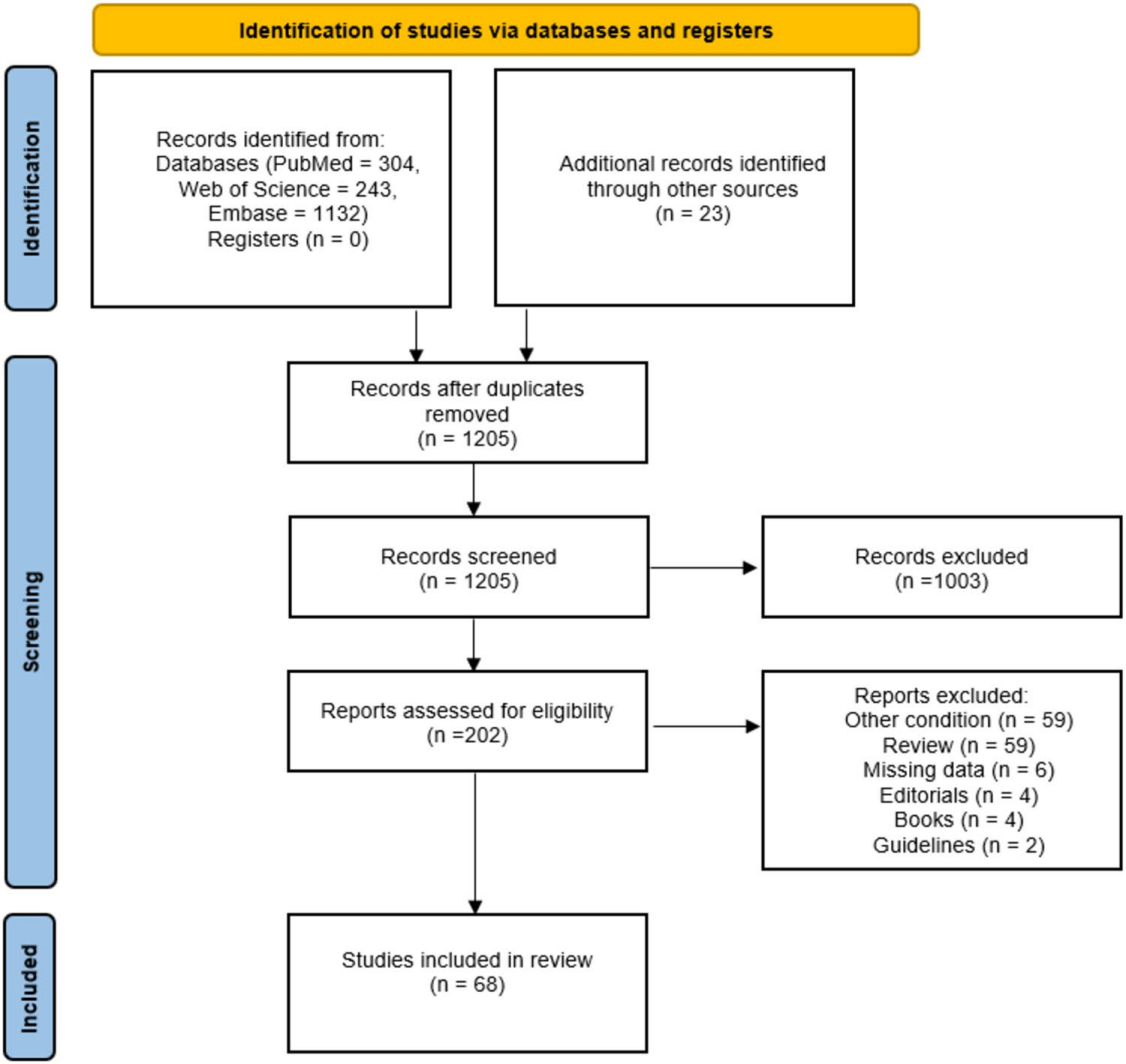
PRISMA flow diagram outlining study selection

From 1205 articles originally identified, 68 articles were included after fulfilling the inclusion criteria (Fig. 1) [9, 12–78], for a total of 136 patients. Most articles were case reports (*n* = 54) and 14 were cases series with a median of 5 patients (IQR 3–9) and a maximum of 14 patients.

The quality assessment confirmed acceptable study quality, with 67 reports (97%) scoring 4 points or more in the modified quality evaluation by Murad et al. (supplementary Table 1). Six and seven of the eight binary questions of the proposed tool were not applicable to this study.

**Fig. 2:**
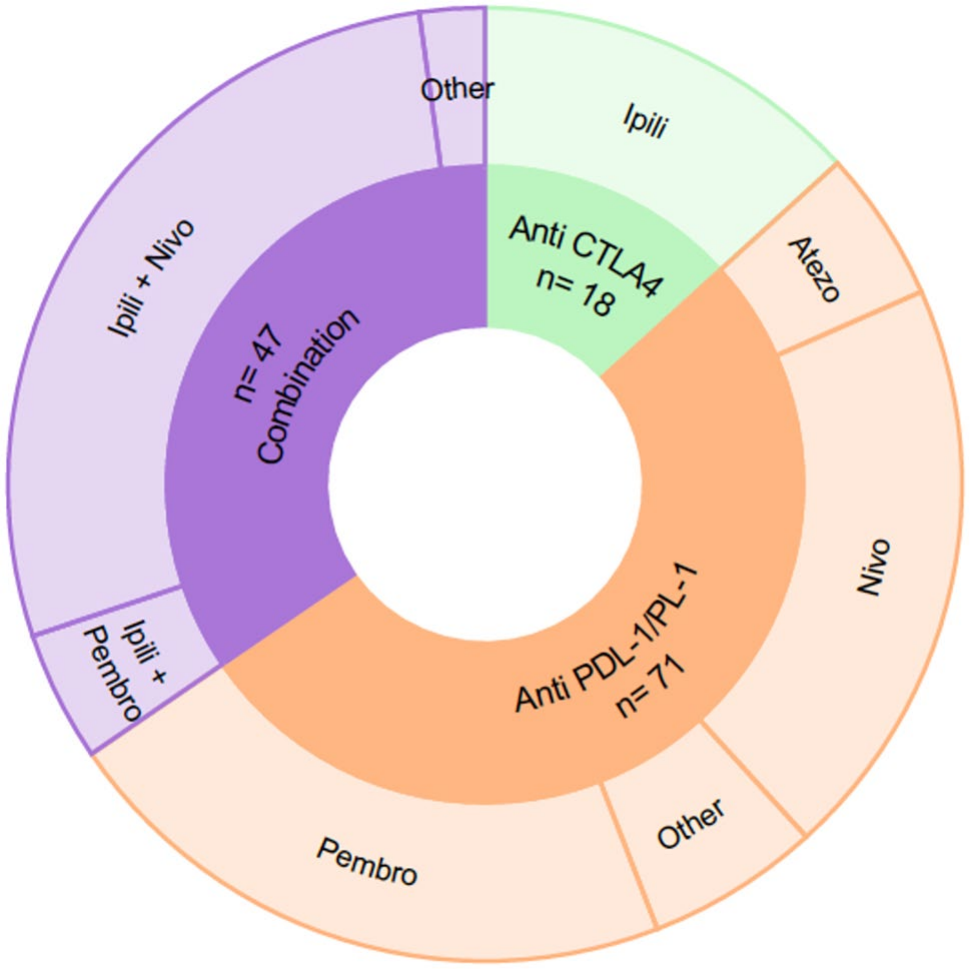
Representation of ICI in the population and their therapeutic targets. *Pembro*, Pembrolizumab; *Ipili*, Ipilimumab; *Nivo*, Nivolumab, *Atezo*, Atezolizumab. Others: Durvalumab (3), Camrelizumab (2), Tremelimumab + Durvalumab (2), Cemiplimab (1), Avelumab (1)

The main characteristics of the population are shown in Table 1. Median age was 63 years (IQR 52–69), 62% (*n* = 78) were male. The main cancers were melanoma in 53% (*n* = 72), non-small cell lung cancer (NSCLC) in 28% (*n* = 22), and clear-cell renal carcinoma (CCRC) in 5.9% (*n* = 8) (supplementary Table 2). Among the selected population, 52% (*n* = 70) were on anti-PD-1/PDL-1, 14% (*n* = 18) on anti-CTLA-4 and 34% (*n* = 48) on a combination of anti-CTLA-4 and anti-PD-1/PDL-1 (Fig. 2). The most common immunotherapy regimens were IPILIMUMAB associated with NIVOLUMAB (31%, *n* = 39), PEMBROLIZUMAB (23%, *n* = 29), and NIVOLUMAB (20%, *n* = 27).

**Table 1:** Demographics, diagnostic work-up, treatment and outcome of 136 patients

Characteristic	*N* = 136
Age	63 (52–69)
Sex	Men (62%)
Cancer type	
Melanoma	72 (53%)
NSCLC	22 (28%)
Others	36 (19%)
Cranial nerve involved	
II	48 (31%)
VI	14 (9%)
VII	51 (33%)
Others	43 (27%)
> 1 nerve involved	17 (12%)
Bilateral involvement	64 (52%)
Concomitant neurological immune-mediated disorders	23 (18%)
Other irAEs (events)	44
ICI-Type	
Anti PD1/PDL-1	70 (51%)
Anti CTLA-4	18 (14%)
Combinaison	48 (35%)
ICI	
Ipilimumab + Nivolumab	39 (29%)
Pembrolizumab	29 (21%)
Nivolumab	27 (20%)
Others	41 (30%)
*Time since first injection (weeks)*	10 (4.3–20)
MRI	96 (83%)
Positive MRI sign	41 (43%)
CST	55 (64%)
Pleocytosis	32 (59%)
Hyperproteinorachia	33 (61%)
Treatment	125 (92%)
Discontinuation	109 (89%)
Corticosteroid	117 (86%)
IgIV	28 (21%)
PLEX	7 (5.1%)
Others	15 (12%)
Outcome irAEs	
Resolution	45 (33%)
Improvement	70 (52%)
Stable	16 (12%)
Worsening	4 (3%)
Tumor outcome under ICI	
Progression	20 (33%)
Response	41 (67%)
Rechallenge	28 (28%)
New irAEs	10 (7%)

*NSCLC* non-small lung cancer, *CSF* cerebrospinal fluid, *PLEX* plasmatic exchange, *IgIV* intravenous immunoglobulin

**Fig. 3:**
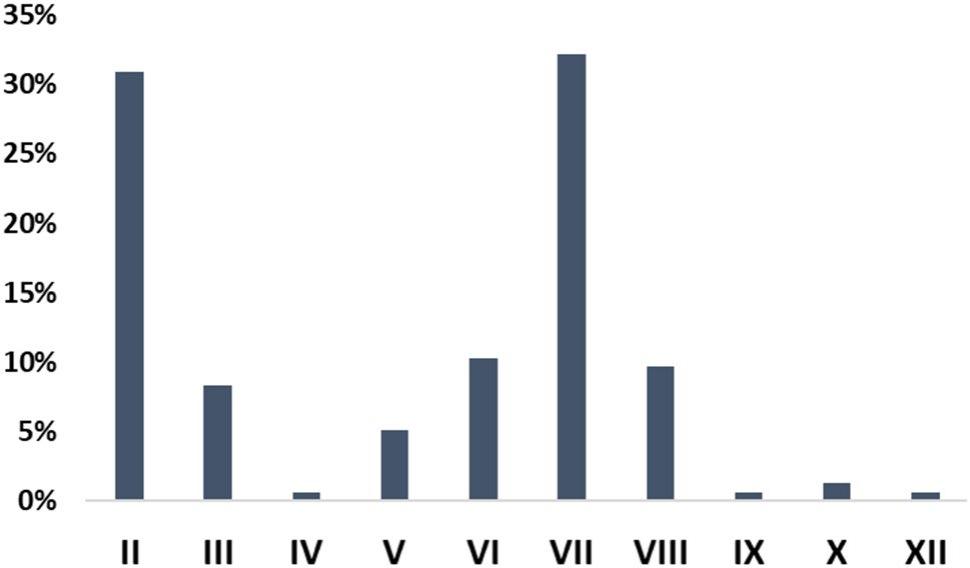
Landscape of the main cranial nerves affected. In total, there were 155 cranial nerves involvement in 136 patients

The most frequent cranial nerve involved was facial nerve involvement (38%, *n* = 51), followed by the optic nerve (35%, *n* = 48), cochleovestibular (12%, *n* = 16), and abducens nerve (10%, *n* = 14) (Fig. 3). Multiple cranial nerve involvement was rare occurring in 12% of patients. 52% had bilateral involvement. There was no association between cranial nerve, as most patients had isolated neuropathy (supplementary Fig. 1). Looking at cranial nerves palsy according to ICI class, we observed that most patients with facial nerve involvement were under anti-CTLA-4, 57% (*n* = 12). In addition, only patients on anti-PD-1/PDL-1 had oculomotor involvement (*n* = 13) (supplementary Table 3). No association was found between specific ICI and CNP-ICI (supplementary Table 4).

In 18% (*n* = 23) of patients, cranial nerve involvement was not isolated, manifesting itself secondarily as a symptom of neurological immune-mediated disorders: Guillain-Barré syndrome (GBS) (54%), Miller Fisher (8.3%) or others type (37%) (supplementary Table 5).

Among the other non-neurological irAEs presented by CNP-ICI patients (20%, *n* = 27) representing 44 events, 21% (*n* = 9) were uveitis, 18% (*n* = 8), hypophysitis, unspecified diarrhea or colitis in 13% of cases (*n* = 6) (supplementary Table 6).

83% of patients had an MRI as part of their workup. Of these, 43% showed contrast or enhancement of the affected nerve. MRI was most sensitive in optic nerve involvement, with 55% positivity (defined by enhancement and/or hypersignal of the nerve) (supplementary Table 7). The CSF shown pleocytosis in 59%, lymphocytic in all cases. Hyperproteinorachia was present in 61% of cases. The presence of pleocytosis was associated with facial involvement (*p* < 0.05) in which 89% had pleicytosis (supplementary Table 8).

**Fig. 4:**
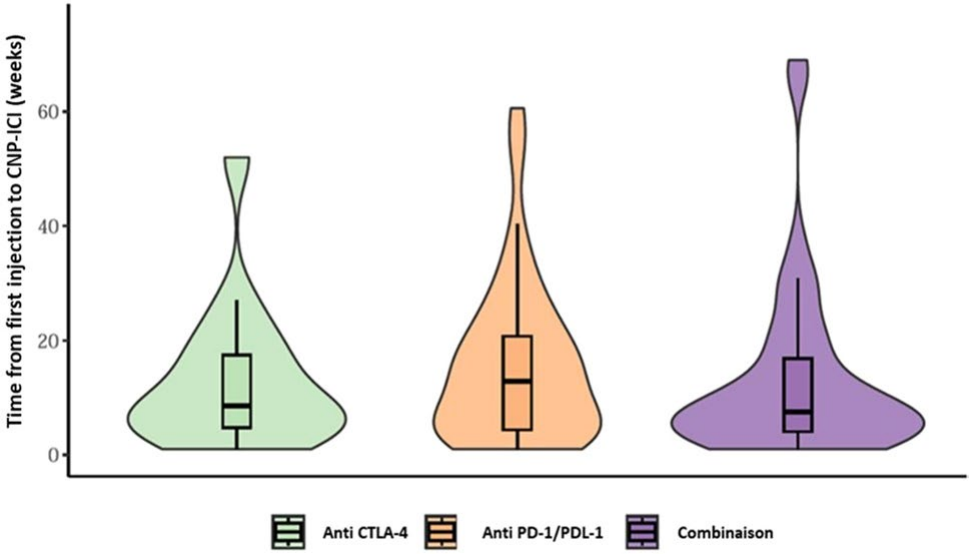
Time between first immunotherapy injection and onset of symptomatology (weeks). Missing data (*n* = 18): Anti PD-1/PDL-1 *n* = 11, Anti CTLA-4 *n* = 2, combination *n* = 5

The median time between the first ICI injection and CNP-ICI was 10 weeks (IQR 4–20), with no significant difference according to the treatment classes (Fig. 4). Furthermore, when looking at specific ICI, none were associated with a significantly different delay (supplementary Fig. 2).

**Fig. 5:**
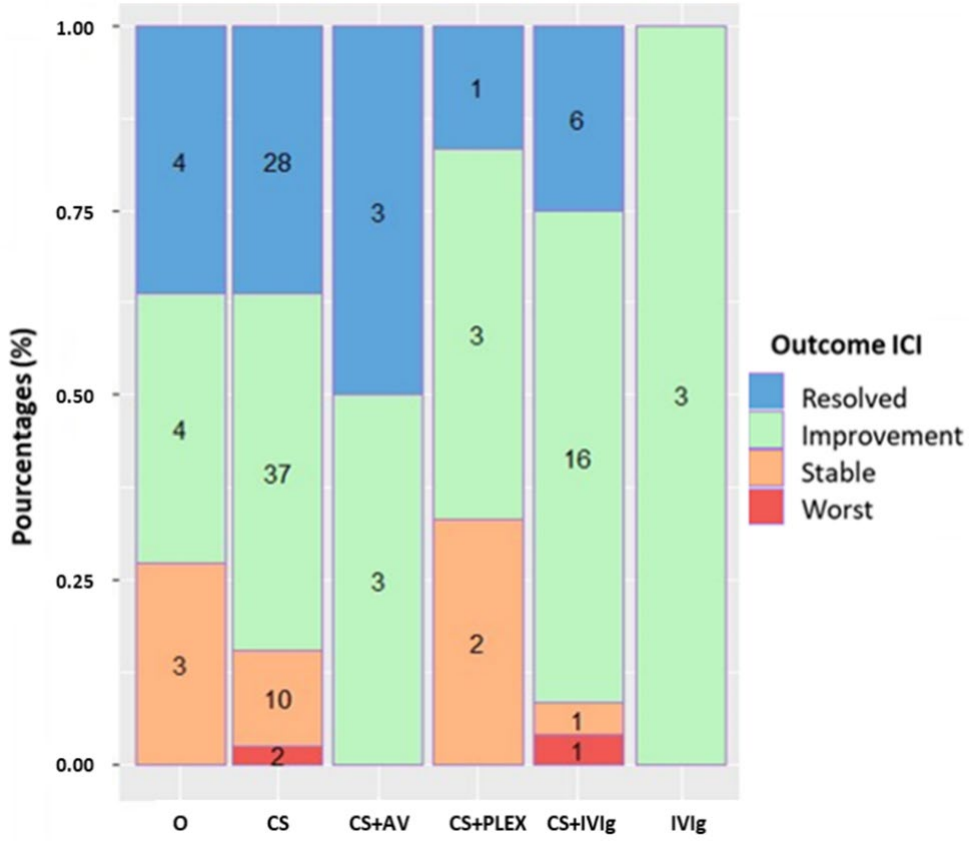
Outcome distribution by main treatments. We didn’t represent the outcome for treatment when a number of patient treated was below 2 (two patients were treated 2 by corticosteroid and rituximab and improved, 2 by mycophenolate mofetil and corticosteroid and improved, 1 patient was treated by antiviral and improved, 1 with only plasmatic exchange and improved, and 1 patient by jak-inhibitor and improved to). *CS* Corticosteroid, *PLEX* Plasmatic Exchange, *IVIg* Intravenous immunoglobulin, *AV* Anti-viral

The first step of CNP-ICI management was immunotherapy suspension in most cases (89%, *n* = 109). Of all patients, 125 (92%) received specific treatment: 117 patients (86%) corticosteroid-based regimen, 28 intravenous immunoglobulin (IVIg) (21%), 9 had antiviral therapy (6,6%), 7 had plasma exchange (PLEX) (5.1%), 2 patients had rituximab, 2 mycophenolate mofetil, 2 infliximab, and 1 patient had Jak-inhibitor. Regarding the outcome, 33% (*n* = 45) of patients had resolution of their toxicity, 52% (*n* = 70) had clinical improvement, 12% (*n* = 16) were stable and 3% (*n* = 4) experienced worsening. Distribution of the outcome was similar between the different cranial nerves affected (supplementary Table 9). No treatment was associated with a more favorable evolution (Fig. 5).

**Fig. 6:**
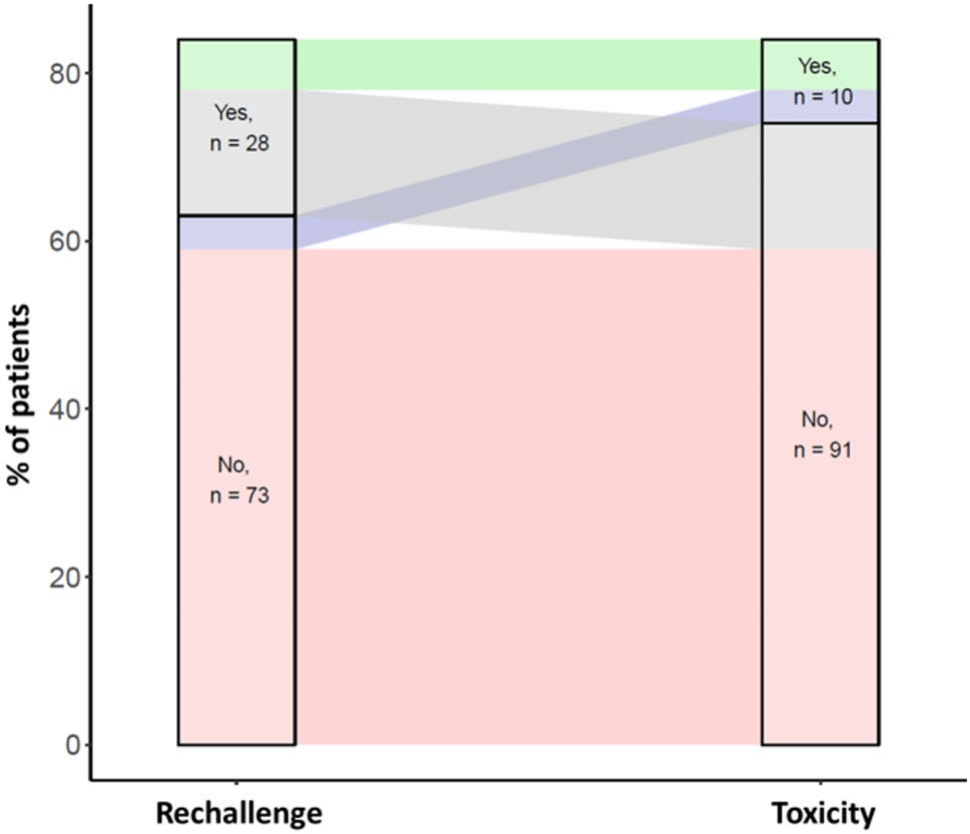
Sankey plot of irAE outcomes with known ICI rechallenge and toxicity status

ICI was resumed in 28 out of 101 patients (28%). Among those patients, 21% (*n* = 6) developed an irAE relapse, including 2/6 patients (33%) presenting the same toxicity (supplementary Table 10). Rechallenge of immunotherapy was associated with new irAE (CNP and new irAE) (*p* = 0.01) (Fig. 6), but we found no significant difference in the evolution of neurological toxicity between patients who suspended immunotherapy and those who continued. 4 out of 73 patients (5%) who did not rechallenge developed a delayed immune-related event.

Only one patient died from neurotoxicity complications (aspiration pneumonia secondary to GBS). Finally, under ICI, most patients (67%, *n* = 41) had an improvement in their oncological status (defined as complete or partial response or stability). We found no association between the type of treatment and tumor progression (supplementary Fig. 3).

## Discussion

In this systematic review of the literature, we describe the clinical and oncological characteristics, treatments, and prognosis of CNP-ICI.

First, the median time between the first injection and toxicity was 10 weeks (IQR 4–20). These observations are consistent with the work of Gougis et al. on VigiBase cases, who found a median delay of 69 days (range 30–152) for the onset of 316 peripheral neuropathies, including CNP-ICI [6]. Secondly, all cranial nerves were affected, with a predominance of facial and optic nerves (38% and 35% respectively) with bilateral involvement in 52%. This contrasts with the rarity of bilateral facial paralysis in Bell's palsy for example, where the autoimmune hypothesis is predominant [79, 80]. Last, we observed that cranial nerve involvement was isolated in most cases.

We found no significant differences in the time to onset of CNP-ICI, neither between classes (PD-1/PDL-1-CTLA-4-combination) or immunotherapy regimen. A key point for clinicians is that, given the rapid onset, poor prognosis, and the fact that the clinical presentation can be purely bulbar, mimicking CNP-ICI, myositis and myasthenia gravis must be ruled out [81–84].

Our findings indicated that MRI revealed hypersignal or enhancement in 43% of cases. While it is important to note that hypersignal or enhancement of the nerve is neither highly sensitive nor specific, this imaging remains particularly valuable for ruling out major differential diagnoses, such as leptomeningeal carcinomatosis [85, 86].

At the onset of CNP-ICI, 89% of patients discontinued their ICIs, and 92% received treatment for CNP-ICI. Plaçais et al. demonstrated that adding corticosteroid therapy to the management of neurological irAEs, compared to discontinuation of ICIs alone, improved neurological outcomes by reducing the occurrence of sequelae [50].

Corticosteroids exert pleiotropic effects on various cells of the immune system. Data suggest their efficacy at the immunological synapse by blocking costimulatory signaling (CD28) or enabling PD-1 expression in the cell [87, 88]. The European Society of Medical Oncology and the American Society of Clinical Oncology guidelines recommend treating patients with grade 2 or higher neurological irAEs with corticosteroids [89, 90]. Tomsitz et al. found that 10.9% of patients in a general irAEs cohort were refractory to corticosteroids, defined as not adequately responding to steroids, experiencing steroid-refractory side effects, or being unable to taper off steroids without the recurrence of side effects [91]. In our study, corticosteroid treatment was associated with an 86% rate of favorable outcomes (resolution or improvement).

Patients who do not respond favorably to corticosteroid therapy may require additional immunosuppressive treatments. There is no consensus on the optimal type of agent to use. In our study, patients received IVIg (19%), PLEX (5%) or mycophenolate mofetil or rituximab (2 patients). We observed a more favorable outcome with the combination of IVIG and corticosteroids (91% favorable evolution) compared to corticosteroids alone (84%), although the difference was not statistically significant. This lack of significance may be attributed to the small sample size of the patient group. In a systematic review of the literature on ICI-induced GBS, IVIg + corticosteroid was associated with 73% clinical improvement [92]. Further studies are needed to confirm the place of IVIg and corticosteroids dual therapy. We found that 29% of those treated with PLEX had stable disease. A key point yet to be defined is the place of PLEX in the management of irAEs. The rationale is based on removing ICI, pathogenic antibodies and others immune effectors of the toxicity [93].

However, pharmacological data suggest that PLEX only partially reduces blood levels of ICI [83].

**Fig. 7:**
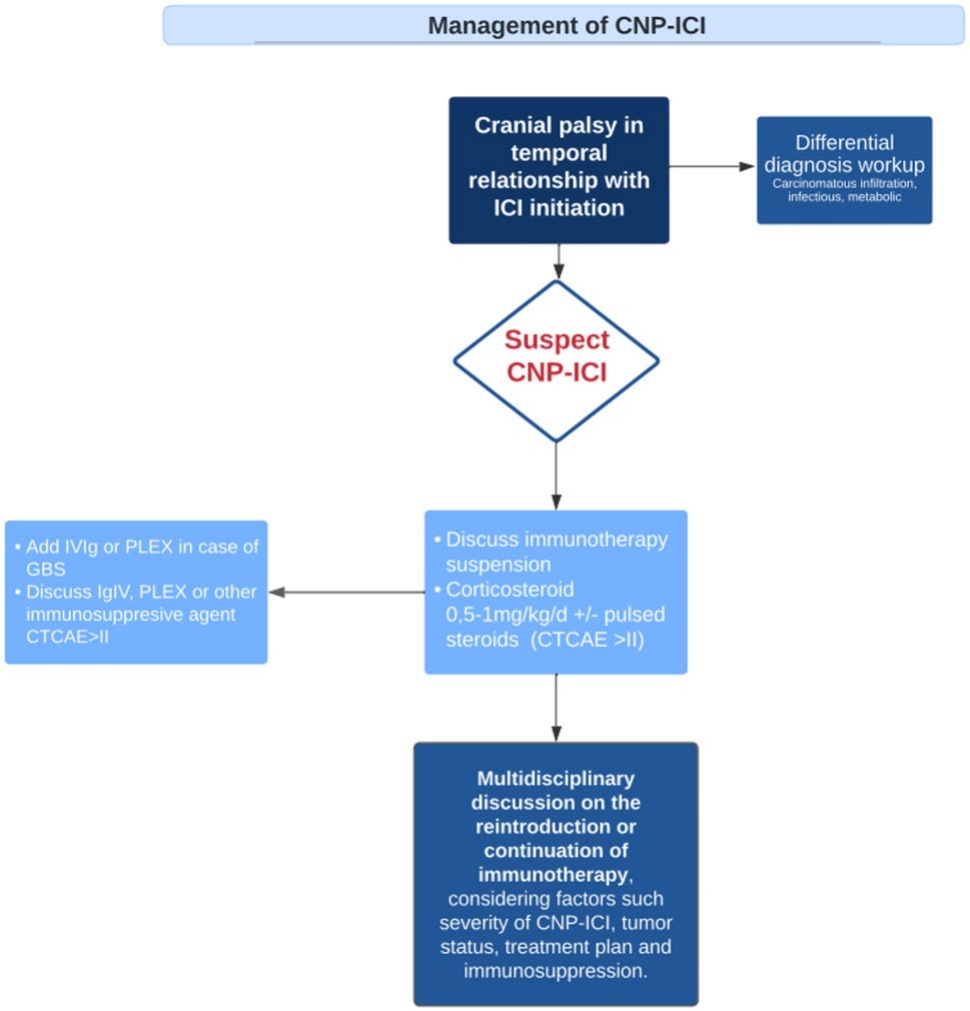
Proposed treatment algorithm for CNP-ICI

In light of these data and the literature review, we suggest a treatment algorithm for CNP-ICI (Fig. 7).

ICI rechallenge was associated with irAE reappearance in 21% of patients in our review. This is in line with a recent meta-analysis, which found recurrent toxicities in 34% [94]. In a large recent cohort, the majority of these recurrent toxicities were previously encountered irAEs [95]. Additionally, new irAEs can occur without rechallenge, manifesting as delayed adverse events even months after the last dose of immunotherapy [96]. These delayed immune-related events can encompass the entire spectrum of irAEs and should be considered as a differential diagnosis even months after discontinuing immunotherapy [97].

Only 1 patient died in our work from complications of CNP-ICI. However, it is crucial to recognize that neurological complications of CNP-ICI can be functionally and vitally life-threatening [98]. In a recent multicenter series from Spain, Fonseca et al. reported an overall mortality rate of 19% attributable to neurological toxicity [99].

In our study, we observed that 67% of patients exhibited a positive tumoral response to ICI. Multiple studies have linked the occurrence of irAEs with favorable tumor responses [100–103]. However, while immunosuppressive agents can effectively address irAEs, they may adversely impact tumor response [104]. This correlation is supported by both animal and clinical models, indicating reduced progression-free survival rates when immunosuppressants are administered early or concurrently with ICI [105–108]. Notably, several investigations have revealed a survival benefit associated with low-grade irAEs compared to high-grade ones [102, 107]. This finding underscores the potential influence of the degree of immunosuppression on patient outcomes, highlighting the critical need to manage this factor to mitigate any adverse effects on tumor progression and treatment efficacy.

The immunopathogenic mechanisms underlying the pathophysiology of irAEs remain to be fully elucidated. The primum movens appears to be a peripheral tolerance breakdown. In animal models, anti-CTLA4 drugs induce major depletion of regulatory T cells, and mouse model of anti-PD-1 deletion resulted in lupus-like proliferative arthritis and glomerulonephritis [109, 110]. This immune hypothesis is supported by the development or relapse of autoimmune disease under ICI, the systemic nature of irAEs, the association between the onset of toxicity and a favorable oncological response, and the good response to immunosuppressive treatments [111]. A recent study identified α-myosin as an autoantigen in ICI-induced myocarditis [112]. Finally, a recent work found evidence supporting an association between thymic alterations and the incidence and severity of ICI-related myotoxicities [113].

One of the strengths of our study is the inclusion of 136 patients, gathered using a methodology based on PRISMA recommendations. We have refined the clinical, therapeutic, and prognostic landscape of CNP-ICI.

A limitation of our study is that diagnoses were made on a presumptive basis in the absence of specific biomarkers, following the exclusion of differential diagnoses. Consequently, cases of CNP-ICI secondary to other causes may have been included. Additionally, we must highlight the significant heterogeneity of our cohort in terms of cancer type, ICI, and response to treatment. Finally, another limitation was the definition of neurological outcomes, which, in the absence of consensus, was determined based on the clinician's judgment.

All included studies were observational with a risk of bias present. The sources of potential bias included limited documentation of patient characteristics and comorbidities, inadequate specification of the inclusion criteria, and lack of multicenter observations.

## Conclusion

We comprehensively reviewed the spectrum, clinical features, and the evolution of cranial nerves induced by ICI treatment. As the utilization of immune checkpoint inhibitors continues to rise, we anticipate that our study will provide valuable insights to clinicians in effectively managing the associated complications of these therapies.

## Electronic supplementary material

Below is the link to the electronic supplementary material.

Supplementary file1 (PDF 584 KB)

## Data Availability

The datasets used and/or analyzed during the current study are available from the corresponding author.

## Appendix

**Table:**

Samuel Pichon	Univ. Lille, CHU Lille, Service de Médecine Interne, Centre de Référence des Maladies Auto-immunes et Systémiques Rares du Nord et Nord-Ouest de France (CeRAINO), F-59000 Lille, France	Designed and conceptualized the study; major role in the acquisition of data; interpreted the data; contribution in statistical analysis; drafted the manuscript
Pauline Aigrain	Univ Lille, CHU Lille, Pôle de réanimation, F-59000 Lille, France	Acquisition of data; interpreted the data; and drafted the manuscript for intellectual content
Charlotte Lacombe	Univ Lille, CHU Lille, Pôle de réanimation, F-59000 Lille, France	Acquisition of data; interpreted the data; and drafted the manuscript for intellectual content
Bruno Lemarchant, MD	Univ. Lille, CHU Lille, Département de neurologie, CRC-SEP, F-59000 Lille, France	Interpreted the data; revised the manuscript for intellectual content
Emannuel Ledoult, MD	Univ. Lille, CHU Lille, Service de Médecine Interne, Centre de référence des Maladies Auto-Immunes et Auto-inflammatoires Systémiques rares de l'Adulte du Nord, Nord-Ouest, Méditerranée et Guadeloupe (CeRAINOM), F-59000 Lille, France. INSERM, Univ. Lille, CHU Lille, U1286-INFINITE-Institute for Translational Research in Inflammation, Lille, France	Interpreted the data; revised the manuscript for intellectual content
Vincent Koether, MD	Univ. Lille, CHU Lille, Service de Médecine Interne, Centre de référence des Maladies Auto-Immunes et Auto-inflammatoires Systémiques rares de l'Adulte du Nord, Nord-Ouest, Méditerranée et Guadeloupe (CeRAINOM), F-59000 Lille, France	Interpreted the data; revised the manuscript for intellectual content
Amélie Leurs, MD	CH Dunkerque, Département de médecine interne et Maladies infectieuses, F-59240 Dunkerque, France	Interpreted the data; revised the manuscript for intellectual content
Ghadi Zebian, MD	Univ Lille, CHU Lille, Pôle de réanimation, F-59000 Lille, France	Interpreted the data; revised the manuscript for intellectual content
David Launay, MD, PhD	Univ. Lille, CHU Lille, Service de Médecine Interne, Centre de référence des Maladies Auto-Immunes et Auto-inflammatoires Systémiques rares de l'Adulte du Nord, Nord-Ouest, Méditerranée et Guadeloupe (CeRAINOM), F-59000 Lille, France. INSERM, Univ. Lille, CHU Lille, U1286-INFINITE-Institute for Translational Research in Inflammation, Lille, France	Interpreted the data; revised the manuscript for intellectual content
Benoit Gachet, MD	Univ. Lille, CH Gustave Dron, Service de Maladies Infectieuses, F-59200 Tourcoing, France Univ. Lille, ULR 2694 METRICS Evaluation des technologies de santé et des pratiques médicales, CH de Tourcoing, F-59000 Lille, France	Interpreted the data; performed statistical analysis; revised the manuscript for intellectual content
Clementine Levy, MD	Univ Lille, CHU Lille, Pôle de réanimation, F-59000 Lille, France	Interpreted the data; revised the manuscript for intellectual content; and supervised the study
