# Supplementary material for: Immune checkpoint inhibitors-associated cranial nerves involvement: a systematic literature review on 136 patients
Source: J Neurol. 2024 Sep 3;271(10):6514–25. doi: 10.1007/s00415-024-12660-2 (PMC11446990; doi:10.1007/s00415-024-12660-2)
Supplement: Supplementary file 1 — Supplementary file1 (PDF 584 KB) [file 415_2024_12660_MOESM1_ESM.pdf]

Supplementary files: Immune Checkpoint Inhibitor-Associated Cranial Nerves Involvement: A Systematic Literature Review on 136 patients

Supplementary figure 1.

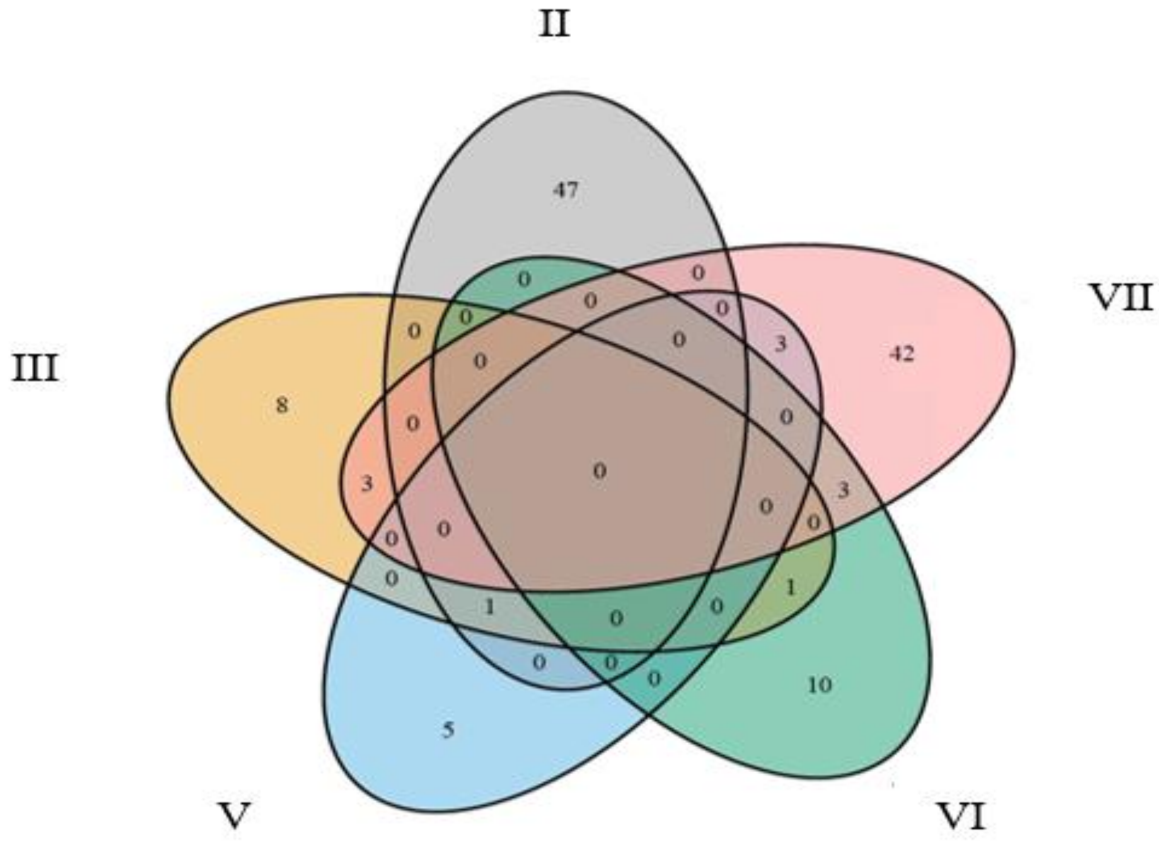

Supplementary figure 1 : Venn diagram showing the co-occurrence of different cranial nerve palsy.

Supplementary figure 2.

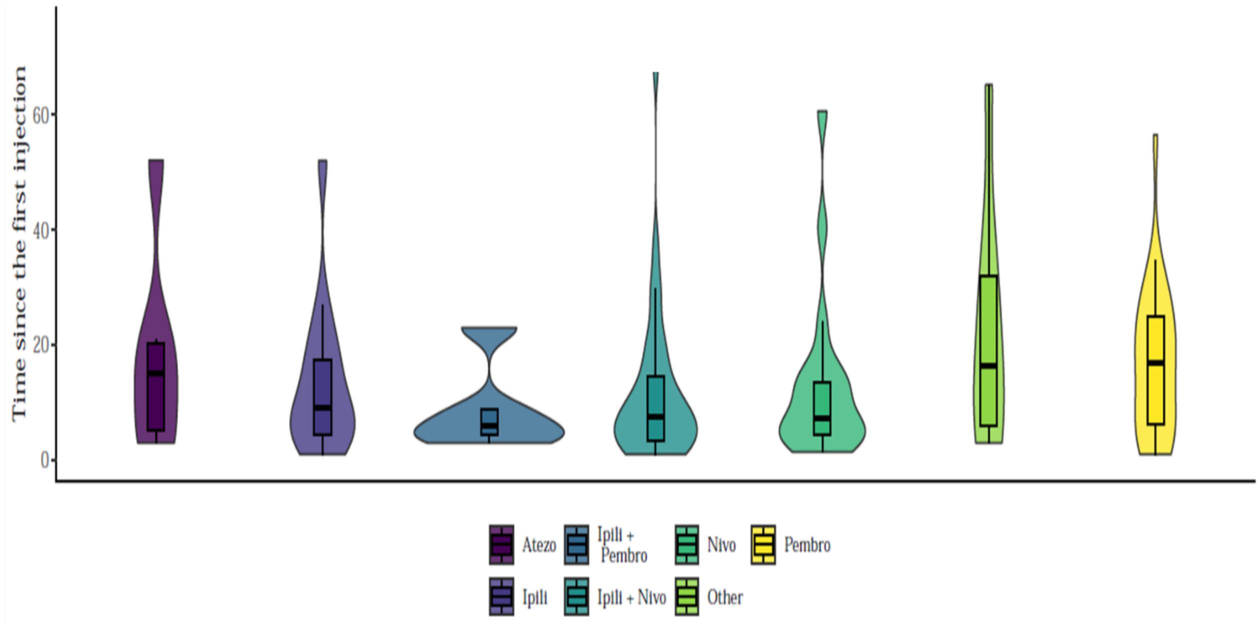

Supplementary figure 2 : Box-plots of the time (weeks) between the first injection of the immunotherapy regimen and the onset of symptomatology.

Missing data (n=18) : Nivolumab n=7, Pembrolizumab n=2, Ipilimumab + pemprolizumab n=1, Ipilimumab n=2, Ipilimumab + Nivolumab n=4, Others n=2.

Pembro ; Pembrolizumab, Ipili ; Ipilimumab, Nivo ; Nivolumab, Atezo ; Atezolizumab.

Supplementary figure 3.

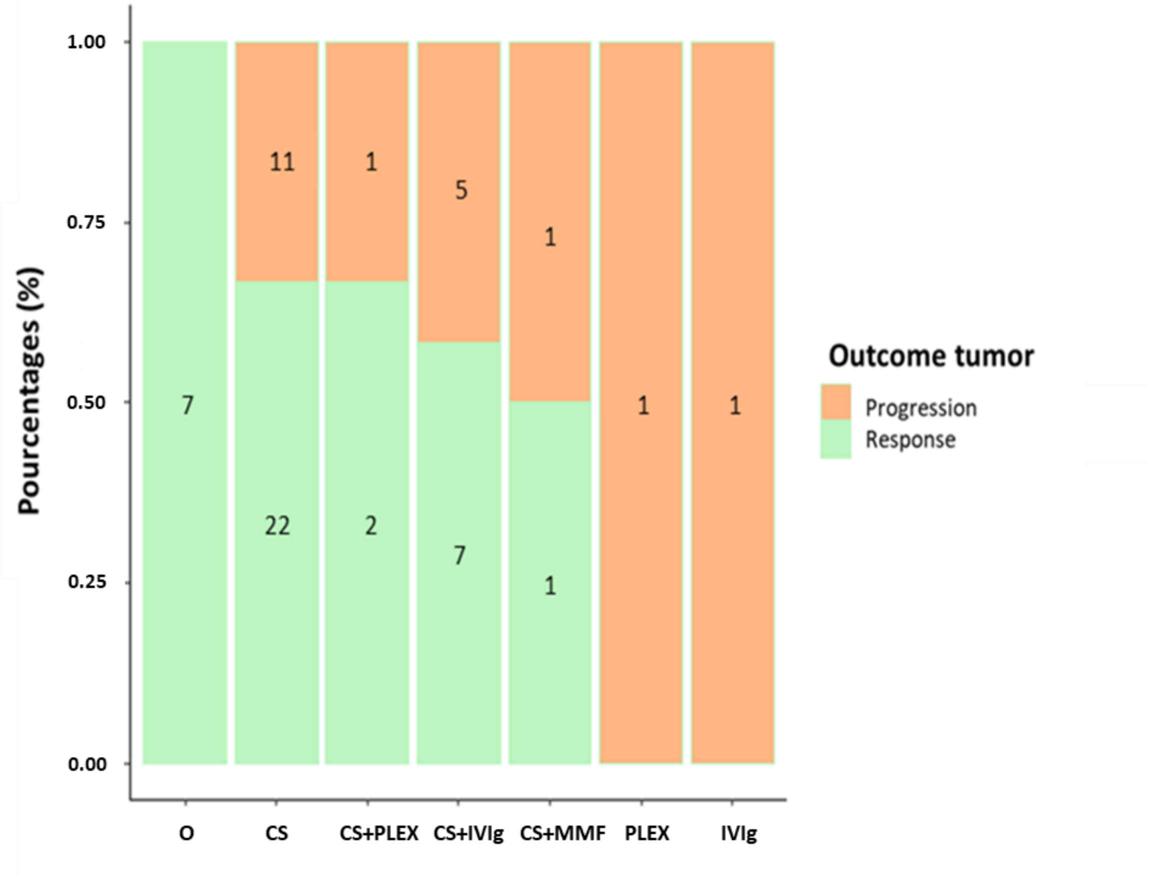

Supplementary figure 3 : Oncological outcome by type of treatment

We have pooled the corticosteroid alone and corticosteroid + antiviral groups in view of the lack of impact of these treatments on tumor survival.

CT : corticosteroid, AV : antiviral, EP : plasmatic exchange, Ig : intravenous immunoglobulin, MMF : Mycophenolate mofetil , R :Rituximab

Supplementary table S1.

	Does the patient(s) represent(s) the whole experience of the investigator (centre) or is the selection method unclear to the extent that other patients with similar presentation may not have been reported?	Was the exposure adequately ascertained?	Was the outcome adequately ascertained?	Were other alternative causes that may explain the observation ruled out?	Was there a challenge/rechallenge phenomenon?	Is the case(s) described with sufficient details to allow other investigators to replicate the research or to allow practitioners make inferences related to their own practice?
Oguri et al[12]						
Xie et al[13]						
Ogawa et al[14]						
McNeill et al[15]						
Wilson et al[16]						
Kichloo et al[17]						
Liao et al[18]						
Zieman et al[19]						
Takemura et al[20]						
Numata et al[21]						
Altman et al[22]						
Vicente-Pascual et al [23]						
Kartal et al[24]						
Willegers et al[25]						
Hou et al[26]						

Wang et al[27]
Wako et al[28]
Diamanti et al[29]
Patel et al[30]
Lemasson et al[31]
Diamantopoulos et al [32]
Vogrig et al[33]
Bolz et al[34]
Zecchini et al[35]
Nowosielski et al[36]
Yost et al[37]
Yuen et al[38]
Green et al[39]
Ong et al[40]
Francis et al[41]
Beninato et al[42]
Yeh et al[43]
Jaben et al[44]
Makri et al[45]
Manousakis et al[46]
Siegel et al[47]
Nishimura et al[48]

Sun et al[49]	
Kao et al[50]	
Plaçais et al[51]	
Farina et al[52]	
Zimmer et al[53]	
Dinoto et al[54]	
Cordes et al[55]	
Duong et al[56]	
Nukui et al [57]	
Noble et al [58]	
Parikh et al[59]	
Kim et al[60]	
Mori et al[61]	
Sengul Samanci et al[62]	

Supplementary table 1 : Quality assessment of included studies.

Supplementary table S2 :

Cancer type	N =136
Melanoma	72 (53%)
Non-small cell lung cancer	22 (16.2%)
Kidney cancer	13 (9.6%)
Small cell lung cancer	6 (4.4%)
Oral, head, and neck cancer	3 (2.2%)
Squamous cell carcinoma	3 (2.2%)
Merkel-cell carcinoma	2 (1.4%)
Bladder cancer	2 (1.4%)
Prostate cancer	2 (1.4%)
Esophageal cancer	1 (0.7%)
Colorectal cancer	1 (0.7%)
Gastric cancer	1 (0.7%)
Hepatic cancer	1 (0.7%)
Hodgkin Lymphoma	1 (0.7%)
Glioblastoma	1 (0.7%)
NA	5 (3.6%)

Supplementary table 2 : Description of cancers in the study population.

Supplementary table S3 :

Cranial nerve/therapeutic class	Anti CTLA-4 (N=21)	Anti PD-1/PDL-1 (N=83)	Combinaison (N=51)
**II**	5 (23%)	27 (33%)	16 (31%)
**III**	0 (0%)	13 (16%)	0 (0%)
**IV**	0 (0%)	1 (1%)	0 (0%)
**V**	1 (5%)	5 (6%)	2 (4%)
**VI**	1 (5%)	7 (8%)	8 (16%)
**VII**	12 (57%)	18 (22%)	20 (39%)
**VIII**	2 (10%)	8 (10%)	5 (10%)
**IX**	0 (0%)	1 (1%)	0 (0%)
**X**	0 (0%)	2 (2%)	0 (0%)
**XII**	0 (0%)	1 (1%)	0 (0%)

Supplementary table 3 : Association between ICI and CNP.

Supplementary table S4 :

Cranial nerve/ICI regimen	IPI + NIVO (N=41)	NIVO (N=36)	PEMB (N=32)	IPI (N=21)	IPI + PEMBRO (N=8)
**II**	11 (27%)	7 (19%)	15 (47%)	5 (24%)	2 (25%)
**III**	0 (0%)	6 (17%)	4 (12%)	0 (0%)	0 (0%)
**IV**	0 (0%)	1 (3%)	0 (0%)	0 (0%)	0 (0%)
**V**	3 (7%)	4 (11%)	1 (3%)	1 (5%)	0 (0%)
**VI**	4 (10%)	3 (8%)	2 (6%)	1 (5%)	2 (25%)
**VII**	19 (46%)	7 (19%)	7 (22%)	12 (56%)	3 (38%)
**VIII**	4 (10%)	4 (11%)	3 (10%)	2 (10%)	1 (12%)
**IX**	0 (0%)	1 (3%)	0 (0%)	0 (0%)	0 (0%)
**X**	0 (0%)	2 (6%)	0 (0%)	0 (0%)	0 (0%)
**XII**	0 (0%)	1 (3%)	0 (0%)	0 (0%)	0 (0%)

Supplementary table 4 : Presentation of the main cranial injuries according to the ICI regimen

ATE = Atezolizumab, IPI = ipilimumab, NIVO = Nivolumab, PEMBRO = Pembrolizumab

Supplementary Table S5:

Non-isolated cranial nerve palsy	N = 23
Guillain-Barré Syndrome	14 (61%)
Miller-Fisher Syndrome	2 (9%)
Chronic Inflammatory Demyeliting Polyneuropathy	1 (4%)
Polyneuropathy	3 (14%)
Multifocal radiculoneuropathy	1 (4%)
Neuronal intermedial filament autoimmunity	1 (4%)
Vasculitis neuropathy	1 (4%)

Supplementary table 5: Description of neurological iAE-ICI associated with CNP-ICI

Supplementary Table S6:

**Others irAEs**	**N = 44 (events)**
Uveitis	9 (21%)
Hypophysitis	8 (18%)
Colitis/diarrhea	6 (14%)
Thyroiditis	4 (10%)
Hepatitis	3 (7%)
Vitiligo	3 (7%)
Rash	3 (7%)
Choroiditis	1 (2%)
Autoimmune hemolytic anemia	1 (2%)
Myelitis	1 (2%)
Renal failure	1 (2%)
Rheumatoid arthritis flare	1 (2%)
Pleural effusion	1 (2%)
Encephalopathy	1 (2%)
Encephalitis	1 (2%)

Supplementary table 6 : Description of other irAEs occurring before or after CNP-ICI

Supplementary Table S7:

**Cranial nerve/MRI**	**Positive MRI**	**Négative MRI**
**II**	2 (55%)	18 (45%)
**III**	3 (37%)	5(63%)
**VI**	2 (40%)	3 (60%)
**VI**	2(25%)	6 (75%)
**VII**	11 (42%)	15(58%)
**VIII**	1 (14%)	6 (86%)

Supplementary Table S7: MRI positivity by nerve (positivity was defined by nerve enhancement and/or hypersignal)

Supplementary Table S8:

Cranial nerve/CSF pleocytosis	Pleiocytosis	No pleiocytosis
**II**	6 (37%)	10 (63%)
**III**	3 (50%)	3 (50%)
**V**	2 (66%)	1 (33%)
**VI**	2(50%)	2 (50%)
**VII**	16 (89%)	2 (11%)
**VIII**	2 (33%)	4 (66%)

Supplementary Table S8: Presence of pleocytosis according to nerve type (positivity was defined by nerve enhancement and/or hypersignal)

Supplementary Table S9 :

Cranial nerve/outcome	Resolution	Improvement	Stable	Worsening
**II**	8 (17%)	30 (62%)	8 (17%)	2 (4%)
**III**	6 (50%)	3 (25%)	2 (17%)	1 (8%)
**V**	2 (25%)	5 (62%)	1 (13%)	0
**VI**	7 (58%)	5 (42%)	0	0
**VII**	18 (44%)	21 (51%)	2 (5%)	0
**VIII**	4 (33%)	4 (33%)	3 (25%)	1 (9%)

Supplementary table 9 : Outcome of main cranial nerves palsy.

Supplementary Table S10 :

Time since last toxicity (weeks)	Rechallenge	ICI	Number of toxicity relapse	Relapse 1 toxicity type	Relapse 2 toxicity type	Relapse 3 toxicity type
24	0		1	Limbic encephalopathy + VI palsy	0	0
16	0		3	Optic neuritis	Optic neuritis	Optic neuritis
28	0		1	Optic neuritis	0	0
5	1	Nivolumab	1	Autoimmune polyneuropathy	0	0
8	1	Nivolumab	2	Hepatitis	Colitis	0
NA	1	Nivolumab + Ipilimumab	2	Optic neuritis	Transverse myelitis	0
8	0		2	Vitiligo	Arthritis	0
6	1	Pembrolizumab	1	Colitis	0	0
4	1	Nivolumab	1	VI neuropathy	0	0
12	1	Ipilimumab	2	Bell's palsy	Hypophysitis	0

Supplementary table 10 : Description of new irAEs after rechallenge or discontinuation
